# Correction: Colon cancer-induced interleukin-35 inhibits betacatenin- mediated pro-oncogenic activity

**DOI:** 10.18632/oncotarget.25959

**Published:** 2018-08-03

**Authors:** Yueqiang Jiang, Yanling Ma, RuiChao Li, JianHai Sun

**Affiliations:** ^1^ Department of Gerontology, Tongji Hospital, Tongji Medical College, Huazhong University of Science and Technology, Wuhan, Hubei Province, China; ^2^ Department of Oncology, the Third People’s Hospital, Wuhan, Hubei Province, China

**This article has been corrected:** The correct author names are given below:

**Yueqiang Jiang and Yanling Ma**

Original article: Oncotarget. 2018; 9:11989-11998. https://doi.org/10.18632/oncotarget.22857

